# Forkhead box O1 and muscle RING finger 1 protein expression in atrophic and hypertrophic denervated mouse skeletal muscle

**DOI:** 10.1186/1750-2187-9-9

**Published:** 2014-09-24

**Authors:** Ann-Kristin Fjällström, Kim Evertsson, Marlene Norrby, Sven Tågerud

**Affiliations:** 1 Department of Chemistry and Biomedical Sciences, Linnaeus University, Kalmar SE-391 82, Sweden

**Keywords:** Acetylation, Atrophy, Denervation, Cytosolic fraction, Forkhead box O, Hypertrophy, MuRF1, Nuclear fraction, Phosphorylation, Skeletal muscle

## Abstract

**Background:**

Forkhead box O (FoxO) transcription factors and E3 ubiquitin ligases such as Muscle RING finger 1 (MuRF1) are believed to participate in the regulation of skeletal muscle mass. The function of FoxO transcription factors is regulated by post-translational modifications such as phosphorylation and acetylation. In the present study FoxO1 protein expression, phosphorylation and acetylation as well as MuRF1 protein expression, were examined in atrophic and hypertrophic denervated skeletal muscle.

**Methods:**

Protein expression, phosphorylation and acetylation were studied semi-quantitatively using Western blots. Muscles studied were 6-days denervated mouse hind-limb muscles (anterior tibial as well as pooled gastrocnemius and soleus muscles, all atrophic), 6-days denervated mouse hemidiaphragm muscles (hypertrophic) and innervated control muscles. Total muscle homogenates were used as well as separated nuclear and cytosolic fractions of innervated and 6-days denervated anterior tibial and hemidiaphragm muscles.

**Results:**

Expression of FoxO1 and MuRF1 proteins increased 0.3-3.7-fold in all 6-days denervated muscles studied, atrophic as well as hypertrophic. Phosphorylation of FoxO1 at S256 increased about 0.8-1-fold after denervation in pooled gastrocnemius and soleus muscles and in hemidiaphragm but not in unfractionated anterior tibial muscle. A small (0.2-fold) but statistically significant increase in FoxO1 phosphorylation was, however, observed in cytosolic fractions of denervated anterior tibial muscle. A statistically significant increase in FoxO1 acetylation (0.8-fold) was observed only in denervated anterior tibial muscle. Increases in total FoxO1 and in phosphorylated FoxO1 were only seen in cytosolic fractions of denervated atrophic anterior tibial muscle whereas in denervated hypertrophic hemidiaphragm both total FoxO1 and phosphorylated FoxO1 increased in cytosolic as well as in nuclear fractions. MuRF1 protein expression increased in cytosolic as well as in nuclear fractions of both denervated atrophic anterior tibial muscle and denervated hypertrophic hemidiaphragm muscle.

**Conclusions:**

Increased expression of FoxO1 and MuRF1 in denervated muscles (atrophic as well as hypertrophic) suggests that these proteins participate in the tissue remodelling occurring after denervation. The effect of denervation on the level of phosphorylated and acetylated FoxO1 differed in the muscles studied and may be related to differences in fiber type composition of the muscles.

## Background

Skeletal muscle normally makes up about 45% of the body mass in humans [1] but is a very plastic tissue responsive to alterations in usage. Muscle inactivity leads to a decrease in mass (atrophy) whereas increased activity leads to an increase in mass (hypertrophy). Such changes in muscle mass are believed to occur as a result of alterations in a delicate balance between pathways regulating muscle protein synthesis and degradation [2]. The Forkhead box O (FoxO) transcription factors FoxO1 and FoxO3 are believed to participate in the regulation of muscle mass since overexpression of these transcription factors has been shown to lead to reduced skeletal muscle mass [3,4].

FoxO transcription factors include the four members FoxO1 (FKHR), FoxO3 (FKHRL1), FoxO4 (AFX) and FoxO6 [5-7]. These are reported to have important roles in e.g. stress resistance and metabolism by regulating the expression of target genes. Examples of environmental stimuli that get translated by FoxO transcription factors into specific gene expression programs include oxidative stress, nutrients and growth factors [8]. In growing cells FoxO proteins are to a high extent located in the cytoplasm [9] since nuclear export is a response to growth signals and nuclear import is a response to stress signals such as oxidative stress [9,10].

One effect of FoxO transcription factors that may be important for the regulation of muscle mass is related to the control of transcription of E3 ubiquitin ligases such as Muscle RING finger 1 (MuRF1) and muscle atrophy F-box (MAFbx, Atrogin1). The mRNA expression of these ubiquitin ligases increase in a number of different atrophic conditions, including immobilization, hind-limb suspension, starvation, glucocorticoid treatment and denervation [11-17]. Similarly the mRNA expression of FoxO1 has been shown to increase in a number of atrophic conditions including denervation [12,15,18]. Constitutively active FoxO1, however, did not increase the expression of MAFbx or MuRF1 in myotubes [19] and transgenic mice overexpressing FoxO1 do not have consistent alterations in MAFbx or MuRF1 levels [4]. FoxO1 has, however, been found to cooperate with the glucocorticoid receptor to synergistically activate transcription of a reporter gene driven by the MuRF1 promoter [20]. The nuclear content of FoxO1 protein has been shown to decrease in human quadriceps muscle after resistance training, associated with muscle growth, and then during a de-training period the amount of FoxO1 protein increased in the nucleus [21].

The functions of FoxO transcription factors are controlled by post-translational modifications such as phosphorylation, acetylation and ubiquitination that influence transport between the nucleus and cytoplasm [22]. FoxO transcription factors can be phosphorylated by a number of different kinases including Akt (protein kinase B). FoxO1 is phosphorylated by Akt on S253, S316 and T24 (mouse FoxO1 sequence). The phosphorylations occur sequentially starting with S253 in the forkhead domain [9]. Following phosphorylation FoxO transcription factors bind to 14-3-3 chaperone proteins and are transported out of the nucleus to the cytoplasm. The 14-3-3 binding masks the nuclear localization signal and this prevents FoxO from returning to the nucleus [10]. In C2C12 myotubes glucocorticoid treatment or removal of growth medium has been shown to decrease the phosphorylation of FoxO1 [3].

FoxO1 can also be acetylated at a number of different sites and acetylation seems to have an inhibitory effect on DNA binding capability but may also stimulate phosphorylation on S253 indicating that acetylation and phosphorylation may work together to control the function of FoxO1 [7,23].

The purpose of the present study was to investigate FoxO1 protein expression, phosphorylation and acetylation as well as MuRF1 protein expression in atrophic (hind-limb) and hypertrophic (hemidiaphragm) 6-days denervated mouse skeletal muscle. The hemidiaphragm muscle becomes transiently hypertrophic for 6–10 days following denervation [24-26] whereas hind-limb muscles atrophy continuously following denervation. The hemidiaphragm of the mouse contains mainly type II muscle fibers with a lower content (about 12%) of type I fibers [27]. The hind-limb muscles used in the present study were anterior tibial muscles that in the mouse are devoid of type I muscle fibers [28] and pooled gastrocnemius and soleus muscles that in addition to type II also contain type I muscle fibers [28,29].

## Results

All results reported are based on data from 7 sets of 8 animals generating 16 denervated anterior tibial muscles with 16 contralateral innervated controls (8 innervated and 8 denervated muscles used for whole muscle protein extraction and 8 innervated and 8 denervated muscles used for preparing separate cytosolic and nuclear fractions), 8 denervated pooled gastrocnemius and soleus muscles with 8 contralateral innervated controls (all used for whole muscle protein extraction), 16 denervated hemidiaphragm muscles, 16 innervated control hemidiaphragms from separate animals (8 innervated and 8 denervated muscles used for whole muscle protein extraction and 8 innervated and 8 denervated muscles used for preparing separate cytosolic and nuclear fractions) and 8 hemidiaphragm muscles from sham operated animals (all used for whole muscle protein extraction).

### Muscle weights

**Figure 1:**
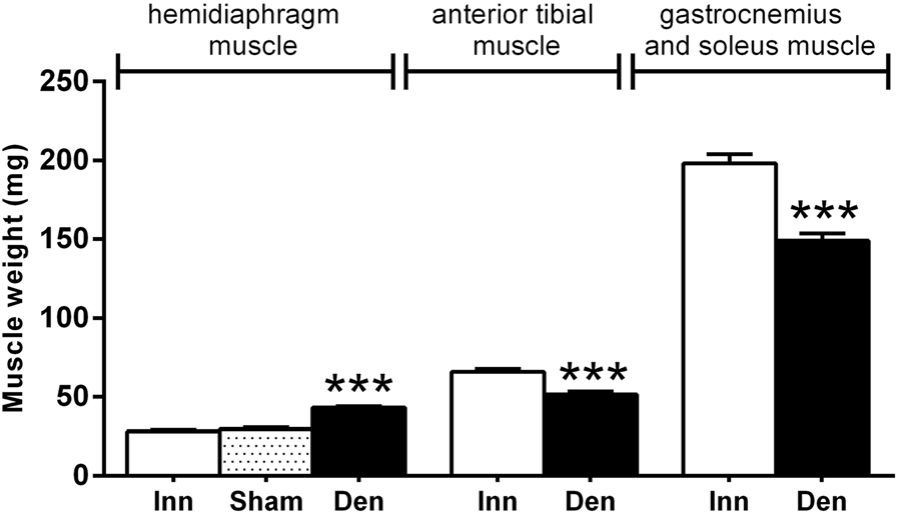
**Muscle weights.** Muscle weights of 6-days denervated (Den) hypertrophic hemidiaphragm muscles, 6-days denervated atrophic anterior tibial and pooled gastrocnemius and soleus muscles compared to innervated (Inn) controls. Mean values ± standard error of the mean. ***p < 0.001, n = 8 muscles per group.

Alterations in muscle weights following 6 days of denervation are illustrated in Figure 1 for muscles used for whole muscle protein extraction. Six days after denervation hemidiaphragm muscles were hypertrophic with a wet weight of 43.3 ± 0.7 mg (n = 8, p < 0.001 versus innervated and sham operated, one-way ANOVA with Tukey’s multiple comparisons test, Figure 1) compared to innervated controls with a wet weight of 28.2 ± 0.8 mg (n = 8) and sham operated control muscles with a wet weight of 29.7 ± 1.0 mg (n = 8). After six days of denervation anterior tibial muscles were atrophic with a wet weight of 51.6 ± 1.8 mg (n = 8), compared to innervated controls with a wet weight of 65.9 ± 1.8 mg (n = 8, p < 0.001, Student’s paired t-test, Figure 1). After six days of denervation pooled gastrocnemius and soleus muscles were atrophic with a wet weight of 149.1 ± 4.4 mg (n = 8), compared to innervated controls with a wet weight of 198.3 ± 5.7 mg (n = 8, p < 0.001, Student’s paired t-test, Figure 1).

Weights of muscles used for preparing separated nuclear and cytosolic fractions were as follows. 6-days denervated hemidiaphragm muscles were hypertrophic with a wet weight of 40.3 ± 1.5 mg (n = 8) compared to innervated controls with a wet weight of 29.6 ± 0.6 mg (n = 8, p < 0.001, Student’s t-test). 6-days denervated anterior tibial muscles were atrophic with a wet weight of 44.7 ± 1.8 mg (n = 8), compared to innervated controls with a wet weight of 60.8 ± 1.9 mg (n = 8, p < 0.001, Student’s paired t-test).

### FoxO1 expression in 6-days denervated atrophic and hypertrophic muscle

**Figure 2:**
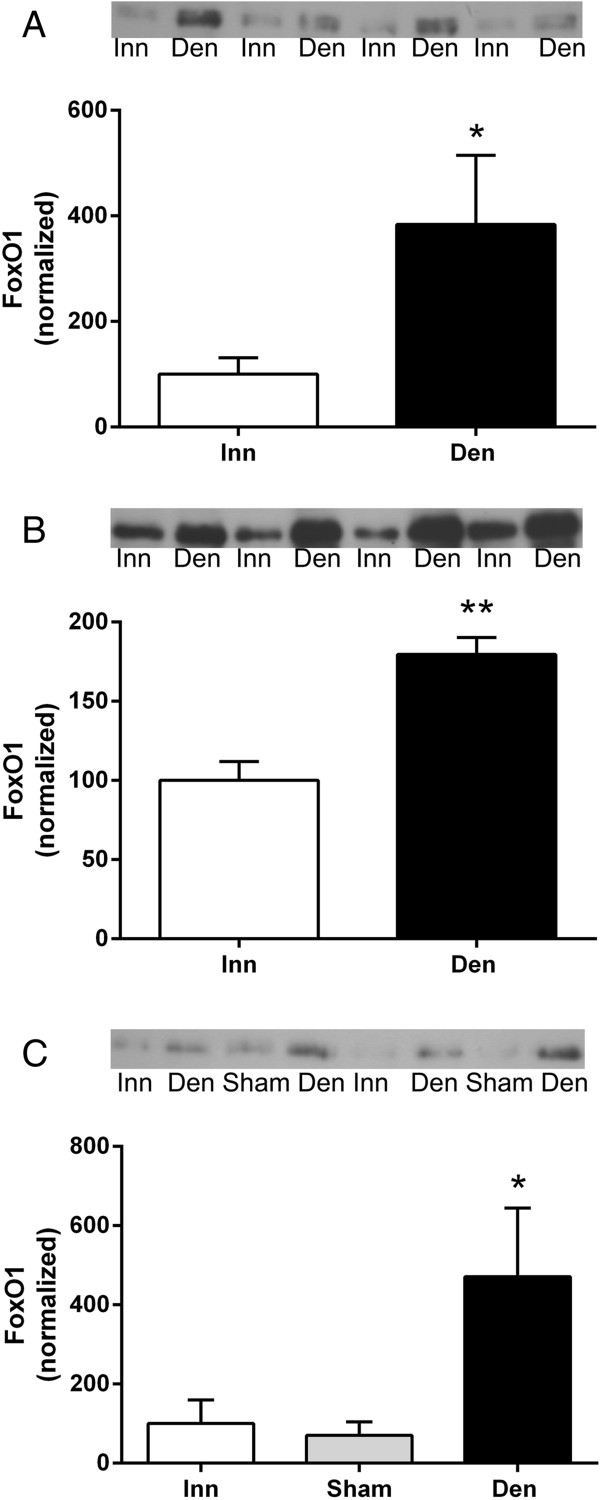
**Total FoxO1 protein expression in 6-days denervated atrophic hind-limb muscles and in 6-days denervated hypertrophic hemidiaphragm muscle.** Total protein expression of FoxO1 in 6-days denervated (Den) atrophic anterior tibial muscle **(A)**, atrophic pooled gastrocnemius and soleus muscles **(B)**, and in hypertrophic hemidiaphragm muscle **(C)** compared to innervated (Inn) and sham operated controls. Representative images of Western blots are shown together with densitometric quantifications. One innervated sample from the respective muscle type was loaded onto all gels as a reference. All samples were measured relative to this reference. The data were normalized to give a mean value of 100.0 in innervated muscles. Mean values ± standard error of the mean. *p < 0.05, **p < 0.01, n = 8 muscles per group.

FoxO1 protein expression increased 0.8-3.7-fold in all 6-days denervated muscles studied, atrophic as well as hypertrophic (Figure 2).

The mean expression level of total FoxO1 protein in anterior tibial muscles was 383.8 ± 131.0 arbitrary units (n = 8) in denervated muscles compared to 100.0 ± 31.1 (n = 8) in innervated muscles (p < 0.05, Student’s paired t-test, Figure 2A). The mean protein expression level in pooled gastrocnemius and soleus muscles was 179.5 ± 10.7 arbitrary units (n = 8) in denervated muscles compared to 100.0 ± 11.9 (n = 8) in innervated muscles (p < 0.01, Student’s paired t-test, Figure 2B). The mean protein expression level in hemidiaphragm muscles was 471.0 ± 173.1 arbitrary units in denervated muscles (n = 8, p < 0.05 versus innervated and sham operated, Kruskal-Wallis test with Dunn’s multiple comparisons test, Figure 2C) compared to 100.0 ± 59.5 (n = 8) in innervated muscles and 70.7 ± 33.6 (n = 8) in sham operated control muscles (Figure 2C).

### FoxO1 phosphorylation in 6-days denervated atrophic and hypertrophic muscle

**Figure 3:**
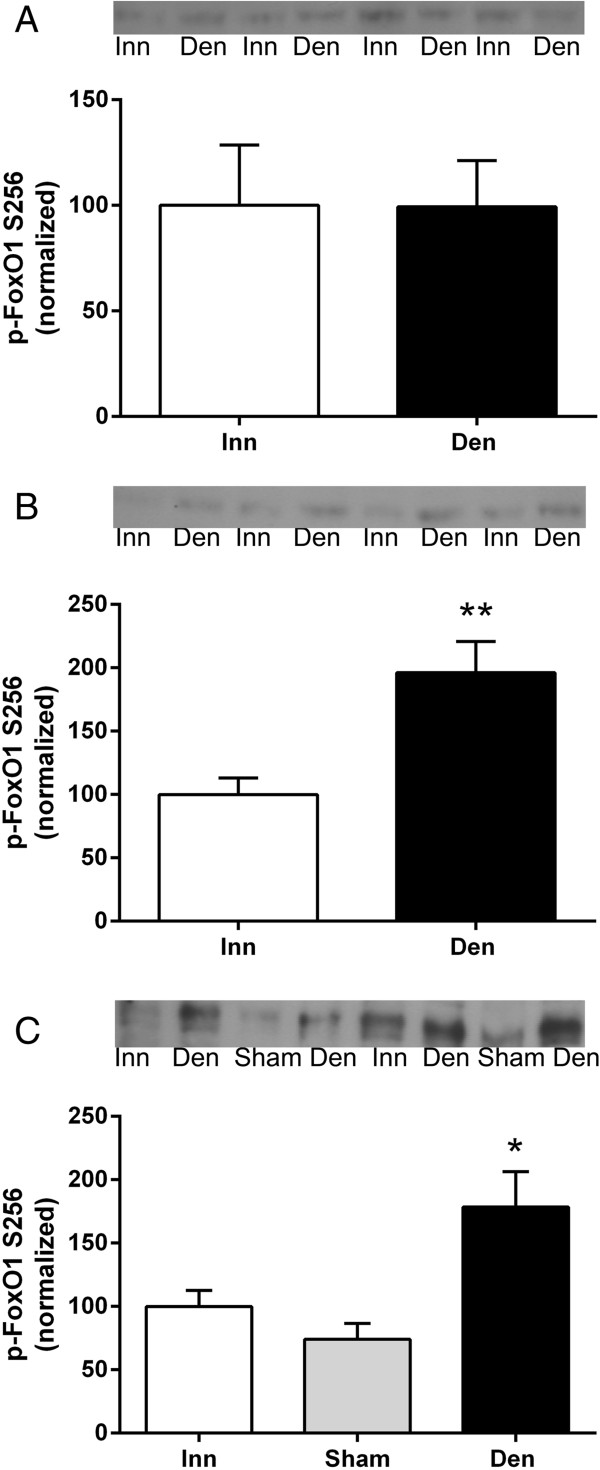
**FoxO1 phosphorylation in 6-days denervated atrophic hind-limb muscles and in 6-days denervated hypertrophic hemidiaphragm muscle.** Expression of phosphorylated FoxO1 in 6-days denervated (Den) atrophic anterior tibial muscle **(A)**, atrophic pooled gastrocnemius and soleus muscles **(B)**, and in hypertrophic hemidiaphragm muscle **(C)** compared to innervated (Inn) and sham operated controls. Representative images of Western blots are shown together with densitometric quantifications. One innervated sample from the respective muscle type was loaded onto all gels as a reference. All samples were measured relative to this reference. The data were normalized to give a mean value of 100.0 in innervated muscles. Mean values ± standard error of the mean. *p < 0.05, **p < 0.01, n = 8 muscles per group.

Levels of phosphorylated FoxO1 were unchanged in 6-days denervated anterior tibial muscle (atrophic) but increased about 0.8-1-fold in 6-days denervated hemidiaphragm (hypertrophic) and in pooled 6-days denervated gastrocnemius and soleus muscles (atrophic, Figure 3).

The mean expression level of phosphorylated FoxO1 in anterior tibial muscles was 99.3 ± 21.9 arbitrary units (n = 8) in denervated muscles compared to 100.0 ± 28.6 (n = 8) in innervated muscles (Figure 3A). The mean expression level in pooled gastrocnemius and soleus muscles was 196.1 ± 24.6 arbitrary units (n = 8) in denervated muscles compared to 100.0 ± 13.1 (n = 8) in innervated muscles (p < 0.01, Student’s paired t-test, Figure 3B). The mean expression level in hemidiaphragm muscles was 178.5 ± 27.9 arbitrary units in denervated muscles (n = 8, p < 0.05 versus innervated and p < 0.01 versus sham operated, one-way ANOVA with Tukey’s multiple comparisons test, Figure 3C) compared to 100.0 ± 12.6 (n = 8) in innervated muscles and 74.0 ± 12.7 (n = 8) in sham operated control muscles (Figure 3C).

### FoxO1 acetylation in 6-days denervated atrophic and hypertrophic muscle

**Figure 4:**
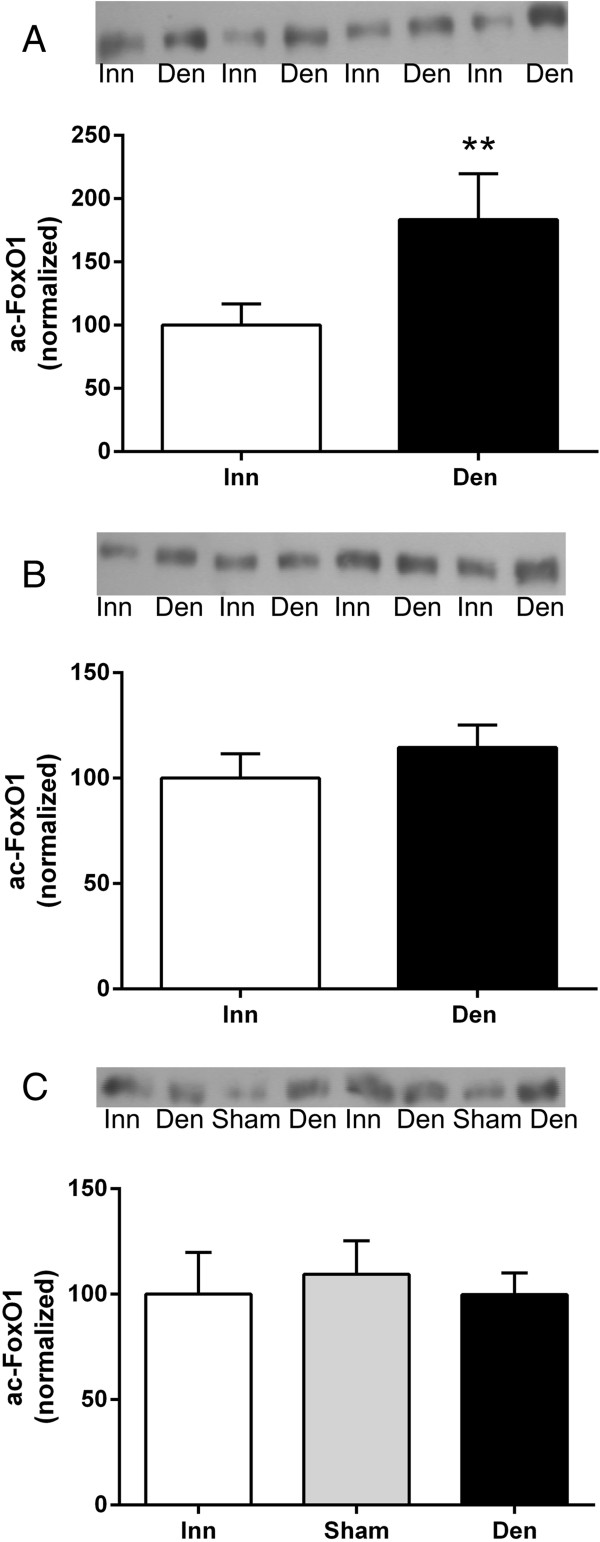
**FoxO1 acetylation in 6-days denervated atrophic hind-limb muscles and in 6-days denervated hypertrophic hemidiaphragm muscle.** Expression of acetylated FoxO1 in 6-days denervated (Den) atrophic anterior tibial **(A)**, atrophic pooled gastrocnemius and soleus muscles **(B)**, and in hypertrophic hemidiaphragm muscle **(C)** compared to innervated (Inn) and sham operated controls. Representative images of Western blots are shown together with densitometric quantifications. One innervated sample from the respective muscle type was loaded onto all gels as a reference. All samples were measured relative to this reference. The data were normalized to give a mean value of 100.0 in innervated muscles. Mean values ± standard error of the mean. **p < 0.01, n = 8 muscles per group.

The level of acetylated FoxO1 increased about 0.8-fold in 6-days denervated anterior tibial muscles (atrophic) but no statistically significant changes were seen in 6-days denervated pooled gastrocnemius and soleus muscles (atrophic) nor in 6-days denervated hemidiaphragm muscles (hypertrophic, Figure 4).

The mean expression level of acetylated FoxO1 in anterior tibial muscles was 183.5 ± 36.2 arbitrary units (n = 8) in denervated muscles compared to 100.0 ± 16.7 (n = 8) in innervated muscles (p < 0.01, Wilcoxon matched-pairs signed rank test, Figure 4A). The mean expression level of acetylated FoxO1 in pooled gastrocnemius and soleus muscles was 114.6 ± 10.6 arbitrary units (n = 8) in denervated muscles compared to 100.0 ± 11.6 (n = 8) in innervated muscles (Figure 4B). The mean expression level of acetylated FoxO1 in hemidiaphragm muscles was 99.8 ± 10.3 arbitrary units (n = 8) in denervated muscles compared to 100.0 ± 19.8 (n = 8) in innervated muscles and 109.5 ± 15.8 (n = 8) in sham operated control muscles (Figure 4C).

### MuRF1 expression in in 6-days denervated atrophic and hypertrophic muscle

**Figure 5:**
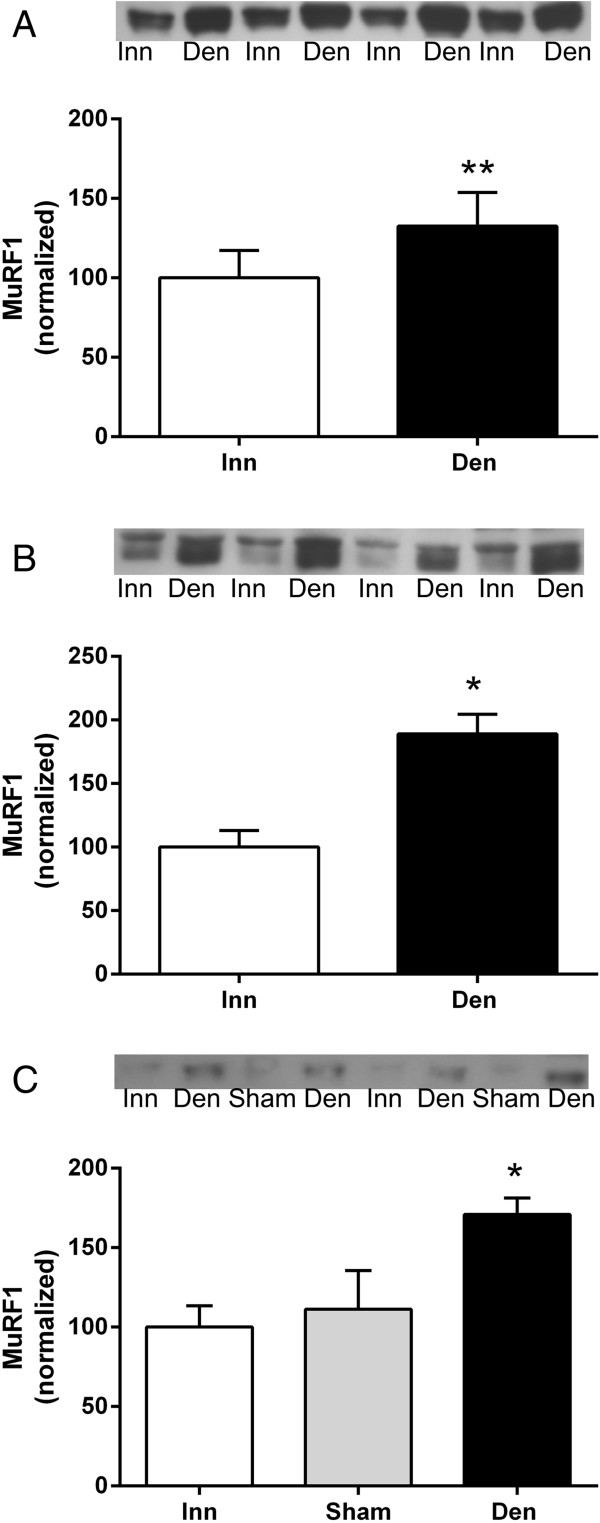
**MuRF1 protein expression in 6-days denervated atrophic hind-limb muscles and in 6-days denervated hypertrophic hemidiaphragm muscle.** MuRF1 expression in 6-days denervated (Den) atrophic anterior tibial **(A)**, atrophic pooled gastrocnemius and soleus muscles **(B)**, and in hypertrophic hemidiaphragm muscle **(C)** compared to innervated (Inn) and sham operated controls. Representative images of Western blots are shown together with densitometric quantifications. One innervated sample from the respective muscle type was loaded onto all gels as a reference. All samples were measured relative to this reference. The data were normalized to give a mean value of 100.0 in innervated muscles. Mean values ± standard error of the mean. *p < 0.05, **p < 0.01, n = 8 (**A** and **C**) or 7 **(B)** muscles per group.

MuRF1 protein expression increased 0.3-0.9-fold in all 6-days denervated muscles studied, atrophic as well as hypertrophic (Figure 5).

The mean expression level of MuRF1 protein in anterior tibial muscles was 132.6 ± 21.2 arbitrary units (n = 8) in denervated muscles compared to 100.0 ± 17.2 (n = 8) in innervated muscles (p < 0.01, Wilcoxon matched-pairs signed rank test, Figure 5A). The mean protein expression level in pooled gastrocnemius and soleus muscles was 189.0 ± 15.6 arbitrary units (n = 7) in denervated muscles compared to 100.0 ± 13.0 (n = 7) in innervated muscles (p < 0.05, Wilcoxon matched-pairs signed rank test, Figure 5B). The mean protein expression level in hemidiaphragm muscles was 170.9 ± 10.4 arbitrary units in denervated muscles (n = 8, p < 0.05 versus innervated muscles, one-way ANOVA with Tukey’s multiple comparisons test, Figure 5C) compared to 100.0 ± 13.3 (n = 8) in innervated muscles and 111.3 ± 24.3 in sham operated control muscles (Figure 5C).

### FoxO1 protein expression and phosphorylation in cytosolic and nuclear fractions of 6-days denervated atrophic anterior tibial muscle

**Figure 6:**
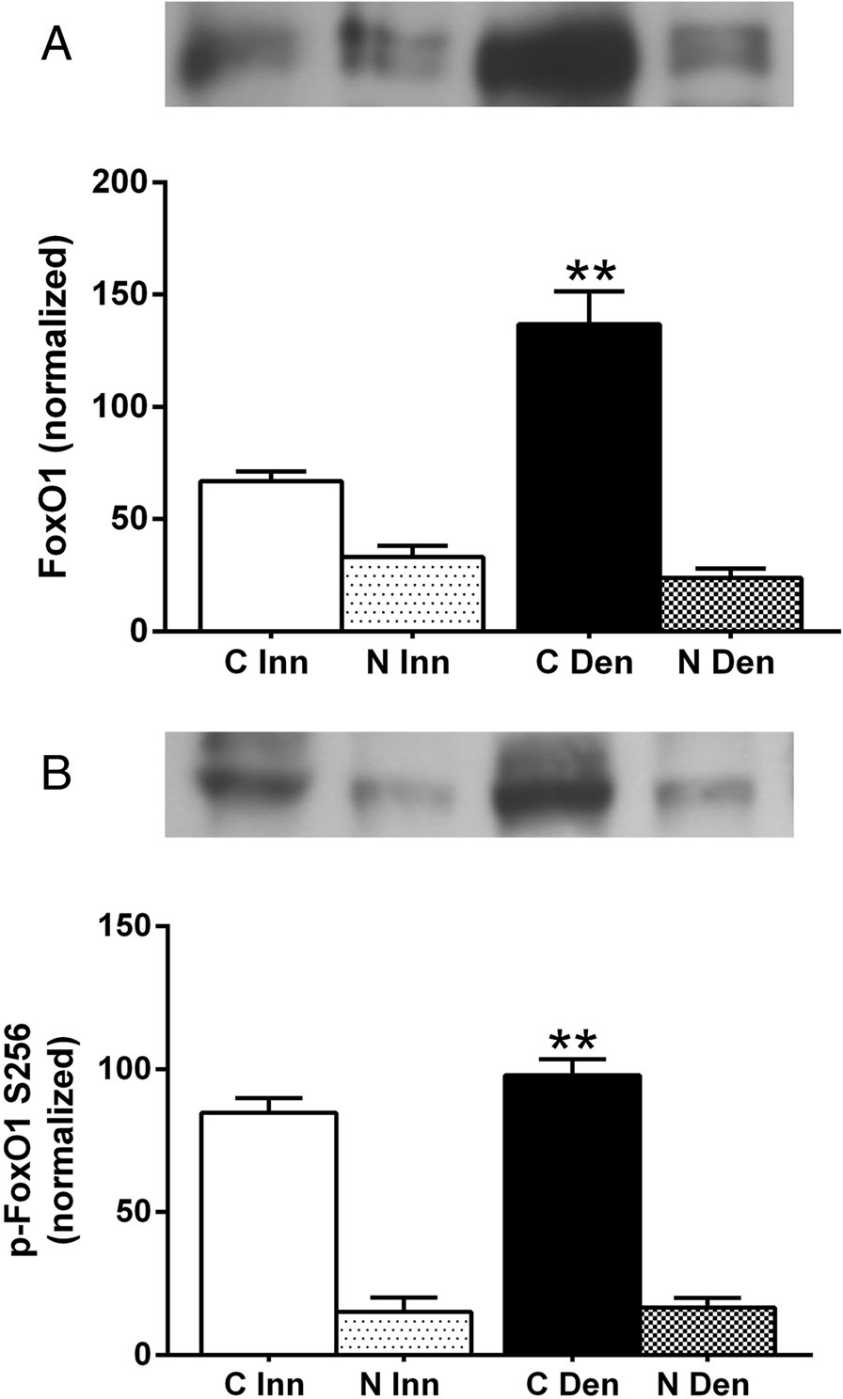
**FoxO1 protein expression and phosphorylation levels in cytosolic and nuclear fractions of 6-days denervated atrophic anterior tibial muscle.** Total FoxO1 protein expression **(A)** and phosphorylation levels **(B)** in cytosolic (C) and nuclear (N) fractions of 6-days denervated (Den) atrophic anterior tibial muscle compared to innervated (Inn) controls. Representative images of Western blots are shown together with densitometric quantifications. One innervated cytosolic sample was loaded onto all gels as a reference. All samples were measured relative to this reference. The data were normalized so that the sum of cytosolic and nuclear signals in innervated muscles will give a mean value of 100.0. Mean values ± standard error of the mean. Statistical comparisons were made between cytosolic fractions of denervated versus innervated muscles and between nuclear fractions of denervated versus innervated muscles. **p < 0.01, n = 8 denervated anterior tibial muscles and 8 contralateral innervated control muscles. Each muscle was fractionated into a cytosolic and a nuclear fraction.

In innervated as well as in 6-days denervated atrophic anterior tibial muscle total and phosphorylated FoxO1 protein were mainly present in cytosolic fractions. Expression increased about 1-fold and 0.2-fold, respectively, in cytoplasmic fractions of 6-days denervated muscles (Figure 6).

The mean expression level of total FoxO1 protein in the cytosolic fraction of anterior tibial muscles was 136.7 ± 14.7 arbitrary units (n = 8) in denervated muscles compared to 66.9 ± 4.4 (n = 8) in innervated muscles (p < 0.01, Student’s paired t-test). In the nuclear fraction the expression level of total FoxO1 protein in denervated muscle was 23.7 ± 4.2 arbitrary units (n = 8) compared to 33.1 ± 5.0 (n = 8) in innervated muscles (Figure 6A).

The mean expression level of phosphorylated FoxO1 in the cytosolic fraction of anterior tibial muscles was 97.9 ± 5.6 arbitrary units (n = 8) in denervated muscles compared to 84.8 ± 5.1 (n = 8) in innervated muscles (p < 0.01, Student’s paired t-test). In the nuclear fraction the expression level of phosphorylated FoxO1 in denervated muscle was 16.7 ± 3.4 arbitrary units (n = 8) compared to 15.2 ± 4.9 (n = 8) in innervated muscles (Figure 6B).

### FoxO1 protein expression and phosphorylation in cytosolic and nuclear fractions of 6-days denervated hypertrophic hemidiaphragm muscle

**Figure 7:**
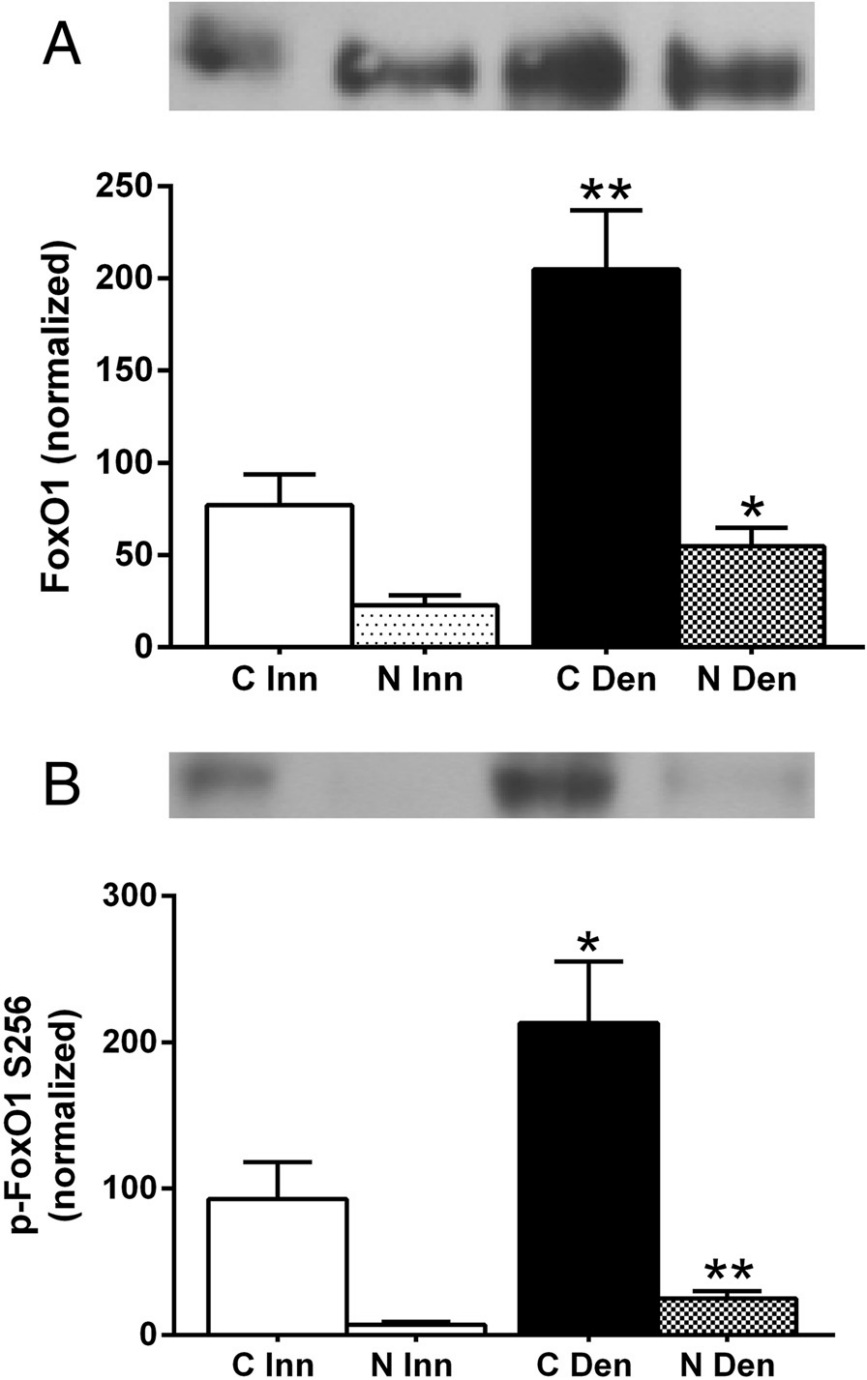
**FoxO1 protein expression and phosphorylation levels in cytosolic and nuclear fractions of 6-days denervated hypertrophic hemidiaphragm muscle.** Total FoxO1 protein expression **(A)** and phosphorylation levels **(B)** in cytosolic (C) and nuclear (N) fractions of 6-days denervated (Den) hypertrophic hemidiaphragm muscle compared to innervated (Inn) controls. Representative images of Western blots are shown together with densitometric quantifications. One innervated cytosolic sample was loaded onto all gels as a reference. All samples were measured relative to this reference. The data were normalized so that the sum of cytosolic and nuclear signals in innervated muscles will give a mean value of 100.0. Mean values ± standard error of the mean. Statistical comparisons were made between cytosolic fractions of denervated versus innervated muscles and between nuclear fractions of denervated versus innervated muscles. *p < 0.05, **p < 0.01, n = 8 denervated hemidiaphragm muscles and 8 innervated control hemidiaphragms from separate animals. Each muscle was fractionated into a cytosolic and a nuclear fraction.

In innervated as well as in 6-days denervated hypertrophic hemidiaphragm muscle total and phosphorylated FoxO1 protein were mainly present in cytosolic fractions. Expression of total FoxO1 protein increased about 1.7-fold and 1.4-fold, respectively, in cytoplasmic and nuclear fractions of 6-days denervated muscles. Expression of phosphorylated FoxO1 increased about 1.3-fold and 2.5-fold, respectively, in cytoplasmic and nuclear fractions of 6-days denervated muscles (Figure 7).

The mean expression level of total FoxO1 protein in the cytosolic fraction of hemidiaphragm muscles was 204.8 ± 32.2 arbitrary units (n = 8) in denervated muscles compared to 77.2 ± 16.6 (n = 8) in innervated muscles (p < 0.01, Student’s t-test). In the nuclear fraction the expression level of total FoxO1 protein in denervated muscle was 54.8 ± 10.1 arbitrary units (n = 8) compared to 22.8 ± 5.5 in innervated muscles (p < 0.05, Student’s t-test, Figure 7A).

The mean expression level of phosphorylated FoxO1 in the cytosolic fraction of hemidiaphragm muscles was 213.3 ± 41.8 arbitrary units (n = 8) in denervated muscles compared to 92.8 ± 25.3 (n = 8) in innervated muscles (p < 0.05, Mann–Whitney test). In the nuclear fraction the expression level of phosphorylated FoxO1 in denervated muscle was 25.2 ± 4.8 arbitrary units (n = 8) compared to 7.2 ± 2.0 in innervated muscles (p < 0.01, Student’s t-test, Figure 7B).

### MuRF1 expression in cytosolic and nuclear fractions of 6-days denervated atrophic and hypertrophic muscle

**Figure 8:**
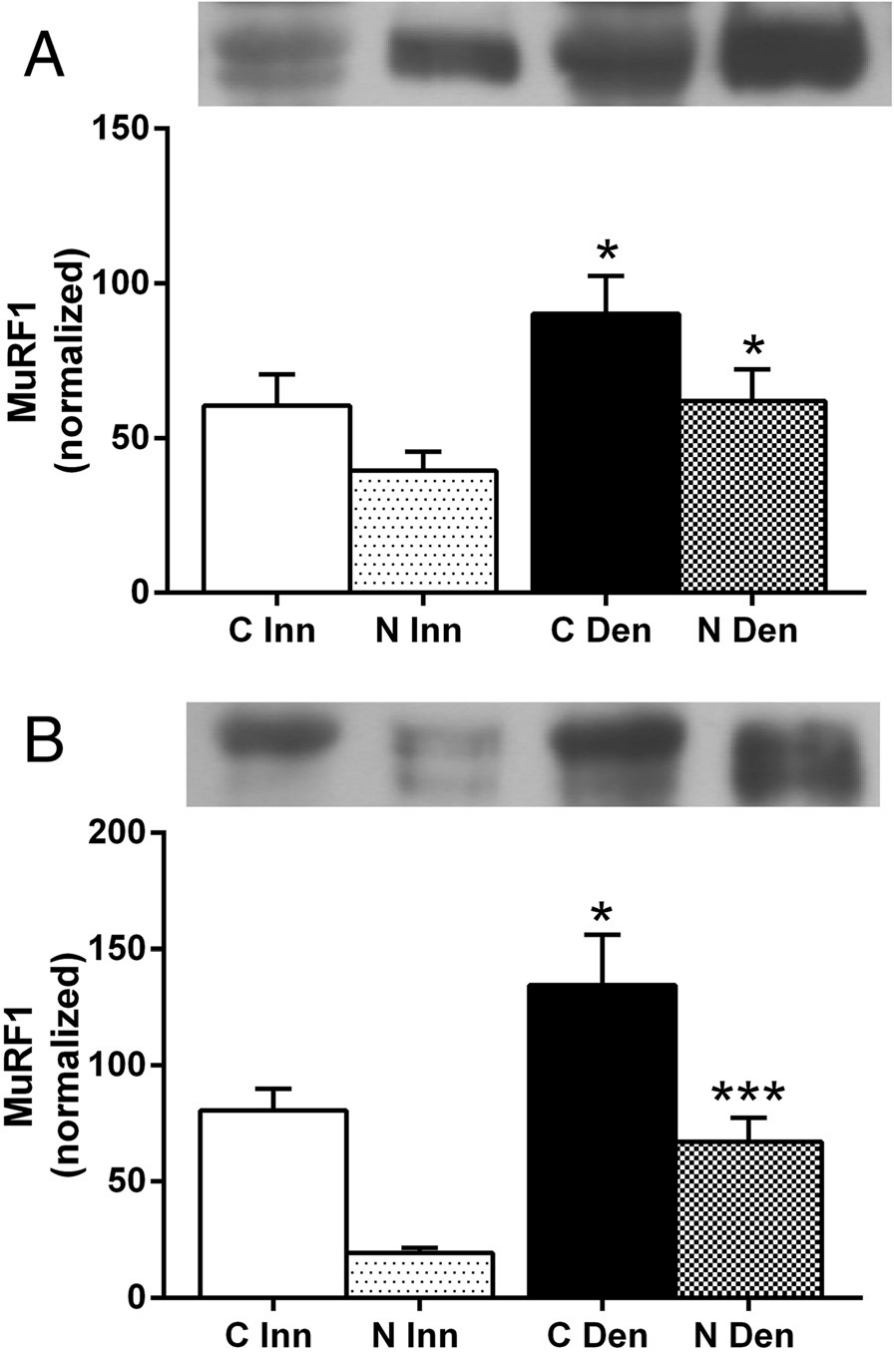
**MuRF1 protein expression in cytosolic and nuclear fractions of 6-days denervated atrophic anterior tibial muscle and in 6-days denervated hypertrophic hemidiaphragm muscle.** MuRF1 expression in cytosolic (C) and nuclear (N) fractions of 6-days denervated (Den) atrophic anterior tibial muscle **(A)** and 6-days denervated hypertrophic hemidiaphragm muscle **(B)** compared to innervated (Inn) controls. Representative images of Western blots are shown together with densitometric quantifications. One innervated cytosolic sample was loaded onto all gels as a reference. All samples were measured relative to this reference. The data were normalized so that the sum of cytosolic and nuclear signals in innervated muscles will give a mean value of 100.0. Mean values ± standard error of the mean. Statistical comparisons were made between cytosolic fractions of denervated versus innervated muscles and between nuclear fractions of denervated versus innervated muscles. *p < 0.05, ***p < 0.001, n = 8 denervated hemidiaphragm muscles and 8 innervated control hemidiaphragms from separate animals. Each muscle was fractionated into a cytosolic and a nuclear fraction.

In innervated as well as in 6-days denervated atrophic anterior tibial muscle and hypertrophic hemidiaphragm muscle MuRF1 protein was mainly present in cytosolic fractions. Expression increased about 0.5-2.4-fold in cytoplasmic as well as nuclear fractions of 6-days denervated muscles (Figure 8).

The mean expression level of MuRF1 protein in the cytosolic fraction of anterior tibial muscles was 90.2 ± 12.2 arbitrary units (n = 8) in denervated muscles compared to 60.4 ± 10.2 (n = 8) in innervated muscles (p < 0.05, Student’s paired t-test). In the nuclear fraction the expression level of MuRF1 protein in denervated muscle was 62.0 ± 10.2 arbitrary units (n = 8) compared to 39.6 ± 6.1 (n = 8) in innervated muscles (p < 0.05, Student’s paired t-test, Figure 8A).

The mean expression level of MuRF1 protein in the cytosolic fraction of hemidiaphragm muscles was 134.5 ± 21.6 arbitrary units (n = 8) in denervated muscles compared to 80.5 ± 9.3 (n = 8) in innervated muscles (p < 0.05, Student’s t-test). In the nuclear fraction the expression level of MuRF1 protein in denervated muscle was 67.1 ± 10.4 arbitrary units (n = 8) compared to 19.5 ± 2.1 in innervated muscles (p < 0.001, Student’s t-test, Figure 8B).

## Discussion

The present study has examined the expression of FoxO1 protein and post-translational modifications of FoxO1 in models of atrophic and hypertrophic denervated skeletal muscle. Most denervated skeletal muscles atrophy but the hemidiaphragm muscle undergoes a transient hypertrophy following denervation possibly as a result of passive stretching due to continued contractions in the contralateral innervated hemidiaphragm [24-26]. The hemidiaphragm of the mouse is composed mainly of type II muscle fibers but also contains about 12% of type I fibers [27]. The hind-limb muscles used in the present study were mouse anterior tibial muscles that are devoid of type I muscle fibers [28] and pooled gastrocnemius and soleus muscles that in addition to type II also contain type I muscle fibers [28,29].

Similar to what has previously been shown for FoxO3 [30-32], and recently also for FoxO1 in atrophic hind-limb muscle [33], the present study shows that the expression of FoxO1 protein is increased in 6-days denervated skeletal muscle. This increase was observed in all denervated muscles studied, atrophic as well as hypertrophic, suggesting that FoxO1 plays a role for denervation changes other than those leading to alterations in muscle mass. One such role may relate to the expression of different myosin heavy chain isoforms. Thus, overexpression of FoxO1 has previously been shown to result in a decrease in type I muscle fibers and a strong reduction in the expression of the slow muscle myosin heavy chain isoform [4]. Similarly, in soleus and gastrocnemius muscles denervation has been shown to reduce the expression of the slow muscle myosin heavy chain isoform [34,35]. Increased FoxO1 expression has also been reported in hypertrophic mouse plantaris muscle following functional overload [36,37].

Phosphorylation of FoxO1 at S256 increased in pooled gastrocnemius and soleus muscles (atrophic with type I fibers) as well as in hemidiaphragm (hypertrophic with type I fibers) but not in unfractionated anterior tibial muscle (atrophic without type I fibers). A small but statistically significant increase in FoxO1 phosphorylation was, however, observed in the cytosolic fraction of denervated anterior tibial muscle. The difference in phosphorylated FoxO1 between denervated anterior tibial and pooled gastrocnemius and soleus muscles might be related to FoxO1 being more readily phosphorylated in type I muscle fibers as suggested by a higher p-FoxO1/FoxO1 ratio in soleus muscle compared to anterior tibial muscle [31]. A statistically significant increase in FoxO1 acetylation was observed only in denervated anterior tibial muscle. A previous study has reported increased acetylation of FoxO3 in denervated anterior tibial muscle but at later times following denervation [30].

FoxO1 protein expression and phosphorylation were also studied in separated cytosolic and nuclear fractions of hemidiaphragm and anterior tibial muscles. In all muscles studied, innervated as well as denervated atrophic and denervated hypertrophic muscles, total and phosphorylated FoxO1 protein were mainly present in cytosolic fractions. In anterior tibial muscle increases in protein expression and phosphorylation were only observed in cytosolic fractions following denervation. In hemidiaphragm total FoxO1 protein, as well as phosphorylated protein, were increased in nuclear as well as in cytosolic fractions following denervation. A previous study has also reported increased nuclear FoxO1 in denervated rat hemidiaphragm although only at early times (1 day) after denervation, but not after 5 days [38].

MuRF1 protein expression has previously been reported to increase in denervated hind-limb muscle [33,39,40]. The present study confirms increased MuRF1 protein expression in denervated atrophic hind-limb muscle but also shows that MuRF1 protein expression is increased in denervated hemidiaphragm muscle at a time point when the muscle is in a hypertrophic state relative to innervated control muscles. Despite the hypertrophic state previous studies on denervated rat hemidiaphragm indicate that from 5 days following denervation protein degradation, as well as protein synthesis, is increased in this muscle [41]. Expression of MuRF1 has been reported to be controlled by myogenin and deletion of myogenin diminishes the expression of MuRF1 in denervated hind-limb muscles [42,43]. Increased expression of MuRF1 in denervated muscle may therefore be a consequence of the increased expression of myogenin that occurs following denervation in hind-limb as well as in hemidiaphragm muscle [44-46]. MuRF1 has also been shown to be preferentially expressed in type II muscle fibers, and also to be preferentially induced in type II fibers after denervation [40,47]. All muscles included in the present study contain type II muscle fibers but the fiber type composition of muscles changes following denervation [48,49]. It is, thus, also possible that the increased MuRF1 expression observed in the present study relates to alterations in fiber types that occur following denervation.

## Conclusions

Increased expression of FoxO1 and MuRF1 in denervated muscles (atrophic as well as hypertrophic) suggests that these proteins participate in the tissue remodelling that occurs in skeletal muscle following denervation. The effect of denervation on the level of phosphorylated and acetylated FoxO1 differed in the muscles studied and may be related to differences in fiber type composition of the muscles.

## Methods

### Animals and muscles

Adult male NMRI mice (Scanbur, Sollentuna, Sweden) were used in this study. The mice were kept in cages with environment enrichment and with free access to a standard laboratory diet and tap water. The animals were anaesthetized by inhalation of isoflurane before surgery and received a subcutaneous injection of buprenorphine (50 μg/kg) for post-operative analgesia. Denervation of either the left hemidiaphragm or the left hind-limb was performed by sectioning and removing a few mm of the phrenic nerve or the sciatic nerve as described previously [50]. Six days after denervation the mice were killed by cervical dislocation. Hind-limb muscles (anterior tibial and gastrocnemius together with soleus) were rapidly dissected, weighed, frozen on dry ice and stored at −80°C. Innervated control hind-limb muscles were collected from the contralateral (right) leg of animals that were denervated by sectioning the left sciatic nerve. Innervated left control hemidiaphragms were collected from separate animals that had received no surgery. To control for this, eight animals used for hemidiaphragm studies went through sham surgery. These animals were anaesthetized by inhalation of isoflurane, had a subcutaneous injection of buprenorphine (50 μg/kg) and a unilateral thoracotomy without touching the phrenic nerve. For dissection of the hemidiaphragm muscle the diaphragm, attached to the rib cage, was quickly removed and placed in cold phosphate buffered saline (PBS) with calcium (2 mM). The left hemidiaphragm was then dissected under a dissecting microscope, blotted dry on filter paper, weighed, frozen on dry ice and stored at −80°C. The experimental manipulations have been approved by the Ethical Committee for Animal Experiments, Linköping, Sweden (permit number: 67–10).

### Protein extraction

Hemidiaphragm, anterior tibial and pooled gastrocnemius and soleus muscles were used for protein extractions. The muscles were homogenized using an Ultra-Turrax homogenizer (Janke and Kunkel, Staufen, Germany) in 1 ml (hemidiaphragm and anterior tibial muscles) or 2 ml (pooled gastrocnemius and soleus muscles) buffer containing 100 mM Tris–HCl, pH 7.6, 150 mM NaCl, 1 mM EDTA, 1% NP-40, 0.1% sodium deoxycholate and 1% Halt™ Protease and Phosphatase Inhibitor Cocktail (Thermo Scientific, Rockford, IL). The supernatant was recovered and the pellet was resuspended in 0.5 ml (anterior tibial and hemidiaphragm muscles) or 1.0 ml (pooled gastrocnemius and soleus muscles) of homogenization buffer and recentrifuged. The supernatants were combined and the protein concentration was determined using the Bradford assay [51].

### Cytosolic and nuclear skeletal muscle fractions

Mouse hemidiaphragm and anterior tibial muscles were used for cytosolic and nuclear protein extraction. The method used was slightly modified from [52] and has been described separately [53]. In brief, muscles that had been stored at −80°C were homogenized using an Ultra-Turrax homogenizer (Janke and Kunkel, Staufen, Germany) in 1 ml low salt lysis buffer (10 mM HEPES, 10 mM KCl, 1.5 mM MgCl_2_, 0.1 mM EDTA, 0.1 mM EGTA, 1 mM dithiothreitol (DTT); pH 7.9 with 1% Halt™ Protease and Phosphatase Inhibitor Cocktail from Thermo Scientific, Rockford, IL). The homogenized tissue was then vortexed (15 s), put on ice (10 min), vortexed again (15 s) and centrifuged (16.000 g for 15 s). The supernatant cytosolic extract was frozen at −80°C for subsequent analyses. The nuclear pellet was resuspended on ice in a high salt nuclear extraction buffer (20 mM HEPES, 420 mM NaCl, 1 mM EDTA, 1 mM EGTA, 1 mM DTT, 25% glycerol; pH 7.9 with 1% Halt™ Protease and Phosphatase Inhibitor Cocktail from Thermo Scientific, Rockford, IL). Four μl of nuclear extraction buffer was used per mg muscle wet weight. Preparations were incubated on ice for 30 min and vortexed (10 s) every 5 min before centrifugation (16.000 g for 6 min). The supernatant nuclear extract was frozen at −80°C for subsequent analyses. Protein determinations for each fraction were obtained using the Bradford assay [51].

### Western blot

Western blots were prepared essentially as described in [50]. Fifteen to thirty μg protein were reduced, denatured and electrophoretically separated on a 12% polyacrylamide gel with a 5.2% polyacrylamide stacking gel on top. Gels were electroblotted onto PVDF Plus transfer membranes (Amersham Hybond-P, GE Healthcare, Buckinghamshire, England) and the membranes were blocked and then incubated with antibodies. Primary antibodies for detecting total-FoxO1 (rabbit monoclonal) (C29H4) [2880] and pFoxO1 S256 (rabbit polyclonal) [9461] were obtained from Cell Signaling Technology (Beverly, CA), MuRF1 (goat polyclonal) [AF5366] was obtained from R&D systems (Abingdon, England) and Ac-FoxO1 (rabbit polyclonal) (FKHR D19) [49437] from Santa Cruz Biotechnology (Santa Cruz, CA). All primary antibodies were used at a dilution of 1/800 – 1/1500. Antibodies were visualized with horseradish peroxidise conjugated secondary immunoglobulin diluted 1/1000 goat anti-rabbit IgG [P0448] and 1/1000-1/10000 rabbit anti-goat IgG [P0449] (Dako, Glostrup, Denmark). The bound immune complexes were detected using the ECL Plus Western blotting detection system and Hyperfilm ECL (Amersham International and Amersham Pharmacia Biotech, Buckinghamshire, England).

### Data analysis and statistics

The expression levels of total, phosphorylated and acetylated proteins were studied semi-quantitatively using data from Western blots. Equal amounts of total, cytosolic or nuclear proteins from innervated or denervated muscles were loaded on the gels. Measured levels of total, phosphorylated or acetylated proteins are expressed without normalization to any specific protein. No loading controls were used and any difference in protein quantifications, pipettings steps, protein transfers etc. are included in the variations of the data sets.

Image analysis was performed using the gel plotting macro of the program ImageJ (Rasband, W.S., ImageJ, US National Institutes of Health, Bethesda, MD, http://rsb.info.nih.gov/ij/, 1997–2007). Results were obtained in uncalibrated optical density units

In order to be able to compare data for whole muscle homogenates run on different gels, one innervated muscle sample (a reference sample) was included in all gels containing samples to be compared to each other. All other samples were measured relative to this reference, the signal of which was set to 100.0 in all gels. In order to more easily compare denervated and innervated muscles all data were finally normalized in such a way that the average signal from innervated muscles became 100.0.

For quantification of protein expression in separated cytosolic and nuclear fractions one of the cytosolic fractions from an innervated muscle was used as a reference sample and was included in all gels. All other samples were measured relative to this reference, the signal of which was set to 100.0. From the amount of protein loaded on gels in relation to the total amount of protein extracted in the nuclear and cytosolic fractions a total cytosolic and a total nuclear signal was calculated for whole muscles. In the final analysis total cytosolic and total nuclear signals were again normalized in such way that the sum of the nuclear and cytosolic signals became 100.0 in innervated muscle.

Data are presented as mean values ± standard error of the mean (SEM). For statistical comparisons of unfractionated hemidiaphragm muscles (innervated, sham operated and denervated) one way-ANOVA was used, followed by Tukey’s multiple comparisons test, for normally distributed data (according to D’Agostino-Pearson omnibus K2 normality test). Statistical significance for data not being normally distributed was determined using the Kruskal-Wallis test with Dunn’s multiple comparisons test. For other comparisons Student’s t-test (paired observations for hind-limb muscles, unpaired observations for hemidiaphragm muscles) was used for normally distributed data. Statistical significance for data not being normally distributed was determined using the Wilcoxon matched-pairs signed rank test (hind-limb muscles) or the Mann–Whitney test (hemidiaphragm muscles).

## Competing interests

The authors declare that they have no competing interests.

## Authors’ contributions

The work presented here was carried out in collaboration between all authors. AKF designed the study and carried out most of the protein expression studies, statistical analyses and drafted the manuscript. KE carried out the studies on nuclear and cytosolic fractions and did the statistical analyses of these. MN and ST conceived of the study, participated in the design, statistical analyses and drafting of the manuscript. All authors have read and approved the final manuscript.
